# Implementing universal HIV treatment in a high HIV prevalence and rural South African setting – Field experiences and recommendations of health care providers

**DOI:** 10.1371/journal.pone.0186883

**Published:** 2017-11-20

**Authors:** Melanie Plazy, Delphine Perriat, Dumile Gumede, Sylvie Boyer, Deenan Pillay, François Dabis, Janet Seeley, Joanna Orne-Gliemann

**Affiliations:** 1 Univ. Bordeaux, Inserm, Bordeaux Population Health Research Center, UMR 1219, Bordeaux, France; 2 Inserm, ISPED, Bordeaux Population Health Research Center, UMR 1219, Bordeaux, France; 3 Africa Health Research Institute, Somkhele, South Africa; 4 Aix Marseille Univ, INSERM, IRD, SESSTIM, Sciences Economiques & Sociales de la Santé & Traitement de l'Information Médicale, Marseille, France; 5 University College London, Division of Infection and Immunity, London, United Kingdom; 6 London School of Hygiene and Tropical Medicine, London, United Kingdom; University of Washington Department of Global Health, UNITED STATES

## Abstract

**Background:**

We aimed to describe the field experiences and recommendations of clinic-based health care providers (HCP) regarding the implementation of universal antiretroviral therapy (ART) in rural KwaZulu-Natal, South Africa.

**Methods:**

In Hlabisa sub-district, the local HIV programme of the Department of Health (DoH) is decentralized in 18 clinics, where ART was offered at a CD4 count ≤500 cells/μL from January 2015 to September 2016. Within the ANRS 12249 TasP trial, implemented in part of the sub-district, universal ART (no eligibility criteria) was offered in 11 mobile clinics between March 2012 and June 2016. A cross-sectional qualitative survey was conducted in April–July 2016 among clinic-based nurses and counsellors providing HIV care in the DoH and TasP trial clinics. In total, 13 individual interviews and two focus groups discussions (including 6 and 7 participants) were conducted, audio-recorded, transcribed, and thematically analyzed.

**Results:**

All HCPs reported an overall good experience of delivering ART early in the course of HIV infection, with most patients willing to initiate ART before being symptomatic. Yet, HCPs underlined that not feeling sick could challenge early ART initiation and adherence, and thus highlighted the need to take time for counselling as an important component to achieve universal ART. HCPs also foresaw logistical challenges of universal ART, and were especially concerned about increasing workload and ART shortage. HCPs finally recommended the need to strengthen the existing model of care to facilitate access to ART, e.g., community-based and integrated HIV services.

**Conclusions:**

The provision of universal ART is feasible and acceptable according to HCPs in this rural South-African area. However their experiences suggest that universal ART, and more generally the 90-90-90 UNAIDS targets, will be difficult to achieve without the implementation of new models of health service delivery.

## Introduction

It is well known that health care providers (HCP) are key actors in the effectiveness of health services, especially regarding HIV care [1, 2]. They are in particular responsible for the technical quality of care (services must be performed according to pre-defined medical standards) and the service quality (how services respond to patients’ expectations and cultural values) [3]. In the fight against HIV infection, HCPs have been in the front line for implementing the successive changes in antiretroviral therapy (ART) initiation eligibility criteria. ART was first recommended by the World Health Organisation (WHO) for HIV-infected patients with moderate and advanced stages of infection, at a CD4 count ≤200 cells/μL in 2002 [4] and a CD4 count ≤350 cells/μL in 2010 [5]. Then, in the last few years, initiating ART earlier in the course of HIV infection was shown to significantly decrease the risk of HIV-related morbidity and mortality [6, 7] and of HIV transmission within serodiscordant couples [8]. As a consequence, the WHO progressively updated its guidelines with ART initiation recommended at a CD4 count ≤500 cells/μL in 2013 [9] and regardless of clinical or immunological stage (i.e. universal ART) in September 2015 [10], as part of the Universal Test and Treat (UTT) strategy [11].

The beliefs, attitudes and experience of experts and HIV specialists towards universal ART have been documented in high-income countries [12–16] where universal access to ART has been available even prior the launch of the 2015 WHO guidelines. Interviewees had good perceptions of the efficacy of universal ART but highlighted the importance of considering patients on a case by case basis, deciding on the best time for ART initiation according to their individual characteristics, related to lifestyle, social support, travels. In sub-Saharan Africa, which carries the greatest burden of HIV, research on access to and delivery of universal ART has mainly been focused on patient perspective, including the investigation of individual and social barriers to HIV care [17, 18]. The perspective of HCPs regarding successes and barriers of offering ART to people early in the course of their HIV infection has not yet been documented, even if it has been identified as essential to inform policy makers in designing service delivery models that are acceptable, efficient and sustainable for the scale up of universal ART [19].

In South Africa, HCPs have provided ART at a CD4 count ≤500 cells/μL in the Department of Health (DoH) HIV programmes since January 2015 [20]. Universal ART was delivered on an experimental basis between 2012 and 2016 within the ANRS 12249 TasP (Treatment as Prevention) trial conducted in KwaZulu-Natal province [21]. As other sub-Saharan countries [22], South Africa has adopted in 2016 the WHO guidelines recommending universal ART as national policy [23]. The number of people on treatment is thus likely to increase from 5.4 million to 7.4 million in 2020 countrywide according to modelling studies [24, 25]. Ensuring that all people living with HIV are able to start ART early in the course of their infection and receive it for life in good conditions will probably be a major challenge for the South African health system overall, and especially for HCPs. The objective of this study is to describe and compare the field experiences and recommendations of clinic-based HCPs working in both the DoH and in the TasP trial clinics regarding the early implementation of universal ART in rural KwaZulu-Natal, South Africa.

## Materials and methods

### Study setting

This study was conducted within Hlabisa sub-district, a rural area of the KwaZulu-Natal province in South Africa, where an estimated 29% of the adults aged 15–49 were HIV-positive in 2012 [26]. The Hlabisa local DoH HIV programme was initiated mid-2004 [27]. It is devolved to 18 primary health care clinics in the sub-district, and is nurse-led, with certain nurses trained for Nurse-Initiated and Managed Antiretroviral Treatment (NIMART) programme since 2011. People can access HIV testing and counselling at any time in these government clinics and receive ART for free if they are eligible, according to the South African national guidelines as follows: at a CD4 count ≤350 cells/μL prior January 2015, then at a CD4 count ≤500 cells/μL until September 2016 when universal ART (no specific criteria for ART initiation) was adopted countrywide.

Between March 2012 and June 2016, the cluster-randomised ANRS 12249 TasP trial was implemented in part of Hlabisa sub-district, under the responsibility of the Africa Health Research Institute (AHRI). This trial aimed to compare the effect of universal ART, initiated in all adults living with HIV regardless of clinical or immunological stage (intervention arm), versus ART initiated according to South African guidelines (control arm), on the reduction in incidence of new HIV infections in the general population [21, 26]. All individuals, aged ≥16 years, reporting being a member of a household of the 22 trial cluster areas, were eligible to participate in the trial. They were offered HIV testing at home every 6 months by fieldworkers; those identified HIV-positive were referred to a cluster-based clinic, set-up especially during the trial period, nurse- and counsellor-led, situated <45 minutes walking from where they lived.

### Study design, study population and sample

A cross-sectional mixed-method survey was conducted among HCPs from both the DoH programme and the TasP trial to investigate their experience in providing early and universal ART, and their perception of the scale-up of UTT [28]. The quantitative component was conducted in April 2016; all TasP and DoH HCP were invited to participate. In this paper, we focus on the qualitative component of the survey, which was conducted in May-July 2016 and consisted of semi-structured individual interviews (IDI) and focus-group discussions (FGD). TasP and DoH HCPs were selected among the staff met during the quantitative component of the study, and based on three main criteria (age, years of experience, high versus low clinic volume) in order to capture diverse professional contexts. Here, the analyses focused on data collected among clinic-based staff, including eight IDIs in TasP (two nurse managers, four nurses and two ART counsellors), five IDIs in the DoH (two government clinic managers and three ART nurses). Two FGDs were also conducted with seven nurses and six ART counsellors, all members of the TasP team (Table 1).

**Table 1:** Study population and sample–Hlabisa sub-district, South Africa, 2016.

		Total population (enrolled in the quantitative component)*	Qualitative study sample	Sample characteristics			
				Interviewee	Gender	Age	Years of experience in HIV care
**HCPs eligible for the quantitative component**							
**TasP**	Clinic-based managers	3 (all enrolled)	2 IDIs	IDI TasP Nurse Manager 01	F	44	11
				IDI TasP Nurse Manager 02	F	39	8
**TasP**	Nurses	19 (15 enrolled)	4 IDIs	IDI TasP Nurse 01	F	35	8
				IDI TasP Nurse 02	F	53	8
				IDI TasP Nurse 03	F	34	6
				IDI TasP Nurse 04	F	71	10
			1 FGD with seven participants	FGD TasP Nurse, P1	M	28	6
				FGD TasP Nurse, P2	F	72	9
				FGD TasP Nurse, P3	F	71	8
				FGD TasP Nurse, P4	F	34	8
				FGD TasP Nurse, P5	F	53	9
				FGD TasP Nurse, P6	F	35	5
				FGD TasP Nurse, P7	F	28	5
**TasP**	ART counsellors	15 (all enrolled)	2 IDIs	IDI TasP Counsellor 01	F	46	16
				IDI TasP Counsellor 02	F	31	9
			1 FGD with six participants	FGD TasP Counsellor, P1	F	58	13
				FGD TasP Counsellor, P2	F	32	6
				FGD TasP Counsellor, P3	F	58	14
				FGD TasP Counsellor, P4	M	29	6
				FGD TasP Counsellor, P5	F	54	14
				FGD TasP Counsellor, P6	F	37	13
**DoH**	ART nurses	51 (40 enrolled)	3 IDIs	IDI DoH Nurse 01	F	47	17
				IDI DoH Nurse 02	F	63	7
				IDI DoH Nurse 03	F	50	6
**Other HCPs**							
**DoH**	Government clinic managers	18**	2 IDIs	IDI DoH Gov. Clinic Manager 01	F	31	3
				IDI DoH Gov. Clinic Manager 02	F	56	17

HCP: health care providers; IDI: individual interview; FGD: focus-group discussion;

TasP: Treatment as Prevention trial; DoH: Department of Health; ART: antiretroviral therapy

F: Female; M: Male; P: Participant

* Total number of HCPs per category at the time of beginning the study in April 2016 (in brackets, number of HCPs enrolled in the quantitative component)

** DoH government clinic managers were not targeted by the quantitative component of the survey

### Data collection

Four local experienced qualitative interviewers carried out the IDIs and FGDs in isiZulu. IDIs lasted for 60 to 90 minutes in English or isiZulu and the FGDs for about 120 minutes in isiZulu. They were held either in a closed meeting room at AHRI (for the TasP staff) or at a place of convenience including within the clinics (for the DoH participants). Structured guides were used for IDIs and FGDs. Each guide was specific to the job position and addressed experiences in providing early ART (DoH) and universal ART (TasP), perceptions regarding the scale-up of universal ART, and perceptions regarding HIV services to increase access to HIV care and treatment. The FGDs and IDIs were audio-taped. Transcription of recordings and translation from isiZulu to English were performed by the interviewers themselves, and all translations were validated independently by the qualitative survey supervisor.

### Analysis process

An inductive approach based on descriptive thematic coding was used. All transcripts were entered into qualitative data analysis software (NVivo version 11, QSR International Pty Ltd., Doncaster, Victoria, 3108, Australia). Codebooks were developed using an iterative process: initial codes matched the predefined research themes (working conditions; attitudes of community members regarding HIV services; future of HIV services), then were expanded with codes reflecting the participants’ own terms and semantics, and discussed within the team, including qualitative interviewers and researchers, for triangulated input.

### Ethics

The study was approved by the Biomedical Research Ethics Committee (BREC) of the University of KwaZulu-Natal on 18 March 2016. All participants voluntarily provided informed written consent.

## Results

### Description of the study sample

Of the 88 HCPs registered as DoH and TasP staff members in April 2016 and eligible to the quantitative component, 73 participated (15 were not present in the clinics; no refusal was recorded). Among them, 24 study participants were purposively selected to participate in the qualitative component; in addition, two DoH government clinic managers were recruited. Overall, 26 participants (24 women and two men) were included; their socio-demographic characteristics are presented Table 1. Briefly, HCPs were between 28 and 71 years old, with a median of 45 years. They had between 5 and 17 years of experience in providing HIV care.

### HCPs are motivated to provide universal ART, which contributes to better health and better care for people living with HIV

#### Early/Universal ART revitalized the community

All TasP and DoH HCPs appreciated initiating people on ART early in the course of the HIV infection (CD4 ≤500 cells/μL in the DoH and regardless of any criteria in the TasP trial), feeling that they were saving people’s lives, participating in reducing their patients’ risk of developing opportunistic infections. HCPs also noted that, beyond individual benefits, early ART had positively impacted on the community. As a consequence of more and more people initiating ART early, they perceived, among the community, increasing confidence and hope in the health system to improve people’s health and fight HIV infection.

“We initiate people [on ART] while they are still fine, so the benefits [of early ART] were helpful. We managed to revive the community […] I think initiating ART early before people become sick, regardless of their CD4 count, has helped the Hlabisa community. Because it wouldn’t be right to find that a whole family is sick, that all die at the same time, and no one is left to take care of the children.” (IDI TasP Nurse 03)

“It has been a while since I last saw a person being transported in a wheel barrow. It means people have hope. They are able to make decisions in time. It is rare to now find a person being bedridden in his/her homesteads. It means that there really is hope, and that we will win this fight.” (IDI TasP Counsellor 02)

#### Early/Universal ART encourages people to seek care

Both TasP and DoH HCPs emphasized that the absence of HIV-related symptoms associated with early ART was a major motivation factor for people to initiate ART as soon as possible, as it contributed to preserve them from HIV-related stigmatization and discrimination from community members.

“[Some patients] approve it (referring to universal ART) as they don’t want to have the symptoms which make it obvious to others that they are HIV positive: symptoms like TB, weight loss and shingles. They like the fact that they recover sooner than the next person noticing that they do not suffer from opportunistic disease. They do not suffer from diarrhoea. People appreciate early ART initiation as other people won’t see those on ART having symptoms.” (IDI TasP Nurse 04)

“If you tell [the patient] that their CD4 count is this much, they would say […] ‘wow it so high!’ […]. The patient appreciates it because you have already taught them that it helps them to not get sick. And so they will not to not be seen [as sick] by people […]. [When] you tell the patient about all the benefits [of initiating ART early], they really like that. Firstly they get surprised by their [high] CD4 count, then, once they know that they will be offered ART [regardless of the high CD4 count], that give them hope.” (IDI TasP Counsellor 01)

Additionally, HCPs witnessed a positive snowball effect of early ART in the communities. Visible health benefits of early ART were encouraging some of those who had been indecisive or reluctant about starting ART, to take action and seek HIV care.

“People who started [early ART] were pioneers into attending the clinics. As others started seeing them getting better, it helped them to feel free to come to the clinics [as well].” (IDI TasP Nurse Manager 01)

#### Early/Universal ART allows for improvements of health care delivery

Both TasP and DoH HCPs appreciated the flexibility in their HIV care practice when dealing with people with higher CD4 counts, who were not urgently in need of ART. They explained that offering ART at high CD4 counts facilitated the relation between HCPs and their patients as asymptomatic people had better capacities of concentration and of understanding the counselling provided compared to the sickest ones. They also enjoyed being able to take time (i.e. several days) to offer more in-depth counselling for these patients and thus better organize their work schedule without rushing for ART initiation.

“It (referring to the change of ART eligibility from CD4 count ≤350 to ≤500 cells/μL) did make a change in [managing] patients, because now we initiate people who are still able to walk around, who are not in bed. It is different from before, when we were initiating people that were emaciated and very weak; it was not easy for them to regain weight and it was taking long. But now it’s easier because the patient is able to walk, is able to answer to all the questions we are asking him/her. It is unlike when the person was very ill and we needed a relative to talk to […] because if the person is too ill, he/she is unable to understand [what we are saying], they are only able to concentrate on their illness […]. [With ART eligibility at 500 CD4 cells/μL], the [patient] queue goes fast.” (IDI DoH Nurse 02)

“With this type of participants [in immediate need of ART], I first need to make them understand that they need to start treatment and that they must come [back to the clinic] the following day [to start treatment]. That participant doesn’t have enough time to understand the situation because I have other duties that I have to do. But I do talk with them and tell them the importance of starting treatment and so on. It is different with participants with who I can have time with [because they have high CD4 count and are therefore not in immediate need of ART]. I can plan their appointment only for counselling […]. Such person [who starts ART at high CD4 count] has time to get detailed information [about ART] as compared to the person who has a low CD4 count [and is in immediate need of ART.” (FGD TasP nurses, P1)

In addition, some HCPs also suggested that the implementation of universal ART could contribute to decrease the burden on the health system, especially related to patients not yet eligible for ART and who were at risk of presenting to the clinic with advanced disease thus requiring hospitalizations.

“I think it (referring to universal ART) can reduce the burden on health care professionals. There are lot of people that are tested HIV positive but because their CD count was above 500 (eligibility criteria for ART initiation at the time of the interview), they would go home and forget about coming back to initiate treatment. [Universal ART] can reduce the number of people we see coming in stretchers and at times they are in Stage 3 and 4. […] This will reduce the number of people who end up being hospitalised as they eventually become severely ill. On the other side this will enslave us. (Laughing). […] we will end up working hard to achieve the target.”(IDI DoH Gov. Clinic Manager 01)

### HCPs perceive challenges in providing universal ART to reluctant patients and without sufficient human resources and equipment

#### Early/Universal ART is offered to people who can be reluctant to initiate ART

Both TasP and DoH HCPs reported that some of their patients were not aware of the rapid changes in the ART landscape and eligibility criteria. Because they lacked knowledge about the evolution of HIV medicine, the benefits of prompt ART initiation upon diagnosis as well as the availability of pills with less side effects than before, those patients were reluctant to initiate ART early in the course of their HIV infection.

“Some [patients] were looking in the past; there were many bad side effects before. Like when people were taking D4T (referring to the ART drug stavudine which has been commonly used in South Africa before 2011), they body structures were changing. So now, they have that fear that if they start taking treatment, they will have big legs or big breasts. That was becoming a concern to them. So your role is to tell them that we know that and we have addressed it. Most of the pills that were causing those problems have been removed.” (FGD TasP Nurses, P3)

Both TasP and DoH HCPs also underlined that they faced specific difficulties in encouraging people with high CD4 count, and especially no HIV-related symptoms, to initiate ART. They acknowledged that HIV disease acceptation could be a long process for those individuals. They were concerned about the fact that some of their patients, and especially the youngest ones, might not see the value of HIV care and ART initiation early in the course of their HIV infection. Some patients feared to engage in taking ART for life. HCPs also noted that some of their patients feared that ART side-effects would make them look sicker than how they truly felt.

“Those who are younger, they see themselves as healthier than others. They look at those with a low CD4 count, saying: ‘oh! I will not go to clinic while I am severely sick and people are holding me’. You see that attitude. […] I feel sad because those persons will end up getting sick. They will decide to start ART only when they are severely sick. Because ART is very strong, the patients may think that it is because of the treatment that is why they are getting worse. I explain to the patients, and ask them to come back so that we can talk.” (IDI TasP Nurse 02)

In this context, HCPs consensually agreed on the key role of counselling to answer the expectations of their new patients, starting from their first clinic visit: allowing them time to claim ownership on the decision of initiating ART early during their infection, and finding the right arguments to make the risk/benefits balance tip in favour of early ART initiation.

“It happens [that people hesitate to initiate early ART], but it’s because of fear of unknown and because when HIV came it came with fear. Some become hesitant saying I will start the treatment and be like my neighbour who is not right and sick […] I don’t feel right but there are skills such as counselling them so they end up leaving with different attitude, knowing the correct way […] they [the patients] respond well on counselling when you talk to them you realise that they just lacked information, they did not know. Once you sit them down and talk to them and tell them how HIV works you notice they now understand and they will say nurse I now know and I’m prepared to do this” (IDI TasP Nurse 03)

#### Early/Universal ART is offered to people who may be more likely to default

Both TasP and DoH HCPs reported overall high ART adherence and retention in HIV care among their patients. However, they also emphasized how having high CD4 count and not feeling sick could compromise HIV disclosure, and thus challenge ART adherence. Based on their experience, some TasP HCPs were further worried that early ART initiation, among people who never felt sick, could lead some of them to forget the treatment benefits, or to not consider their health as a priority, which could result in treatment interruption, and ultimately emergence of ART resistance and complexification of HIV care management.

“The dangers of early ART initiation is that, after some time, people stop taking ART. If they start treatment without ever being ill, they default and then develop resistance. You cannot guarantee that that these people will adhere to their treatment and not get tired of taking the treatment. They can say ‘I have never been ill, I am now tired of this treatment’. When people default and start taking treatment again, they develop resistance [to their ART regimen]. And then we will have a lot of regimen 2, and we do not like that.” (IDI TasP Nurse 03)

“If they [referring to people with high CD4 count] start their treatment early, it can cause them not to adhere because they see they are healthy. They think that they can move to other places like Durban or Gauteng, and that they can even stop taking their treatment. Some will come back [to the clinics] when they are really sick. Good adherence is more found in people who started treatment when their CD4 count was low, when they really needed to take treatment, than in those who have a high CD4 count, who have never been sick and for who it is really easy to dropout from taking treatment. They would just say ‘I was not around for two weeks, my ARVs were finished’.” (FGD TasP Nurses, P2)

HCPs again identified personalized quality counselling as a cornerstone for the success of ART adherence among people initiating early: discussing disclosure to trigger sustainable personal support and regularly reminding the role of ART in maintaining one’s high health status.

“Counselling is a continuous thing. You don’t do it in one day. In a sense that even with a participant who is taking treatment, it’s a day to day counselling. Even if his/her adherence is good, you encourage him/her differently. Even that participant needs a counselling that is directed to him/her and according to his/her behaviour.” (FGD TasP Nurses, P2)

#### Early/Universal ART could suffer from human resources and equipment shortage

All HCPs were in favour of making universal ART available in the area that they serve. Nevertheless, most of them pointed out several foreseen programmatic challenges of large-scale implementation of universal ART. As universal ART implies an increased patient volume receiving care, HCPs feared a decline in the quality of care in the local government clinics, in the absence of adequate efforts from the health care management in terms of human resources and health equipment. HCPs were concerned about clinic staff experiencing too much workload and wearing themselves out. And about patients suffering from longer waiting queues, receiving less individualized counselling, facing drug shortages and delayed lab tests results, and thus loosing motivation for HIV care and treatment. HCP overall highlighted the risks of quality deterioration and lessening of trust in both the HCP-patient and the HCP-health care management relationships.

“Even if [DoH staff] can be ready [to implement universal ART], the staff shortage will hinder their readiness. […] A nurse can’t work well when over loaded and it compromises the quality of care. […] You would end up just dishing the medication […] and missing a lot in the process. You would end up missing a person with unsuppressed viral load, […] or with abnormal blood results or missing the normal health education. You would end up focusing on the [patient’s] queue in the bench, and realize that you have so many patients to attend.” (IDI TasP Nurse Manager 02)

“The concept [of universal ART] is good. The problem is: will it be sustainable? Hum… we have seen periods in the Hlabisa district, when [clinics] run short of treatment […]. Then, patients are like yoyos. They will be on treatment, but what about the following day? They will not be. Because of ordering, or they have changed tenders. I think that is the frustrating part when it comes to treatment.” (IDI TasP Nurse Manager 01)

“Shortage of treatment makes me sick. Because when patients start taking treatment, we tell them that: ‘Don’t forget it. Don’t miss the pill’. So if there is no treatment [that we can give them], how can the patients trust us? How can they believe that they will survive if there are days when they do not take their treatment?” (IDI DoH Nurse 03)

### HCPs suggest opportunities for successful scale up of universal ART

All HCPs were in favour of scaling-up universal ART in the government clinics of the area that they serve, while emphasizing that a successful implementation would require the healthcare management to command drastic changes in the current health system.

#### Efforts in constituting a wide and efficient workforce

First, HCPs urged health care management to adequately match human resources needs to promote quality health care and optimize patient management. They also advised on acknowledging the specific challenges of universal ART delivery and acceptability, therefore adequately training the nursing workforce to provide an adequate and personalized counselling to their patients.

“[Providing] HIV care [to someone] is like caring [for any other] person. When they come to the clinic, you should be able to attend to all their needs, not only issuing them HIV treatment. Everything, including the social aspects [of HIV care]. […] So if the work load is increased (with the implementation of universal ART), I don’t think the nurses will be able to attend to the social needs [of their patients]. They will only focus on the queue, and just issue [people] tablets without knowing other problems the patients may also have.” (IDI TasP Nurse 03)

#### Efforts in offering a diversity of ART supply strategies

HCPs suggested that several ART supply strategies could support a successful large-scale universal ART programme. They referred to services that they either knew of, experienced or witnessed in their work environment.

First, they advised on driving health facilities to be more flexible in providing ART to their patients: in opening clinics on Saturdays and systematically offering 3 months refill for stable patients (i.e. who are virally suppressed) in order to reduce the number of required ART pick-up visits and decrease the risk of non-adherence. Some DoH HCPs also experienced allowing community members (e.g. community caregivers (CCGs), directly-observed therapy (DOT) supporters, relatives) to pick up the drugs on behalf of patients, and such initiative was appreciated by community members who had difficulties to access the clinics to take their treatment.

“People that give us difficulties [in ensuring they keep adhering to ART] are those who work in far areas, such as truck drivers. […] But since we have started this new system of giving them three months of treatment, I think it’s getting better now because they won’t run out of treatment. We also give them a chance of sending their relatives to collect treatment for them, if their CD4 count remains controlled […] And when they are back from work they can come anytime for check-up. We tell them to keep the date for blood taking, and for all other tests to come at least once a year.” (IDI DoH Nurse 02)

“Yah, there is a change right now it (referring to workload) has decreased again because of the chronic clubs; it is a programme for patients that are viral load suppressed and who adhere very well to their treatment. I’ve been taking to the fields where people are being given treatment by the CCGs. […] The patients are happy about with the change because sometimes they do not have money to come here so to collect their treatment. So it helps a lot for the CCGs to collect their treatment as they only come sometimes twice a year for review of their bloods.” (IDI DoH Gov. Clinic Manager 02)

Then, both TasP and DoH HCPs highlighted the importance of offering a combination of HIV care services, including ART delivery for stable patients, with the implementation of community-based services in addition to the health facilities. Moving beyond the clinic walls, directly in the community or in people’s home, could indeed facilitate access to HIV care and treatment for older people, or those who live far from the clinic and/or who can’t afford transportation costs.

“They are a few old women and men [who attend clinics for HIV care] […] If people could be tested [for HIV] at home and be given ART at home, this would decrease the number of people on ART who default. Taking a taxi to go to the clinic is expensive […]. If it could be known that on this day, we (referring to HIV HCPs) are visiting these people, they would then get their medications. That will ensure that no government funds will be wasted when people get sick because they default and therefore need serious medical care from the hospital, which costs money.” (IDI TasP Counsellor 01)

Finally, TasP HCPs reported positive experiences in using phone calls and small text messaging (SMS) to encourage people identified HIV-positive within the trial in going to the clinics for ART initiation and follow-up. But one nurse questioned the ability of the DoH health system to implement such strategy, considering the nigh number of patients to contact.

“Another thing that I liked [in the TasP trial], even though it was a bit of work, is that we were following our participants. For example, if you find that if a participant is no longer coming to the clinic [for HIV care], you know that you have his/her phone number, then you can call him/her. Or you can track [at home] the participant with all this TasP staff (referring to the dedicated workforce who visited people’s home to encourage them to initiate or adhere to ART). But I am not sure if the government can be able to use that system because he will have a huge number of participants.” (FGD TasP Nurses, P6)

#### Efforts in integrating universal ART in a more comprehensive approach

Both TasP and DoH HCPs highlighted that ART initiation, especially among people with high CD4 count, could be positively influenced by the provision of HIV and ART information prior to clinic visit (including information on universal ART). They acknowledged how community mobilization, such as community-awareness campaigns (i.e. roadshows, as implemented within the TasP trial), or media mobilization (through TV or radios), could help to increase HIV and ART education as well as change cultural beliefs in the community. TasP staff also underlined the value of home-based HIV counselling and testing with fieldworkers who provided first HIV counselling.

“The challenge that they (referring to the DoH nurses) might face [in implementing universal ART in their clinics] are people’s cultural believes, [fear of] stigma and [HIV status] denial. I will encourage them (referring to DoH staff) to focus on awareness as people do not change because they do not have the information. People do not understand what is in them (referring to the HIV virus) or how does it affects them. [But,], if people have a clearer picture, they do comply. They (referring to the DoH staff) must also […] work hand in hand with the leaders and heads in the area. If they strengthen those rapports, it will be easy for them to approach people”. (IDI TasP Nurse Manager 02)

“Some people listen to media, read pamphlets. Then they come to us aware of what is going on. […] This made it easy [for us to work], because when they come to us they already know most things.” (IDI DoH Nurse 01)

“Another thing that makes it easy is if s/he was referred by a trained fieldworker, who has counselling, who provided enough counselling for one to decide to come to the clinic. That makes it easy. When you start counselling that person, you see that they know a lot already and they are committed to start with us” (FGD TasP Counsellors, P1)

Additionally, TasP and DoH HCPs also suggested that universal ART acceptability in the community would benefit from involving actors from the community in the programme implementation, for example during awareness campaigns. Trusted community leaders such as religious representatives and ward counsellors (officially elected heads in South Africa) could provide reassurance and guidance to community members regarding their HIV care pathway; some people living with HIV could also stand up as expert-patients to testify on their experience and act as role models for those who have uncertainties or doubt regarding HIV care.

“I think there is a role that they (referring to community leaders) have to play [in the implementation of universal ART]. They should let the community know, they should preach the gospel that if you know that you are HIV-positive, you should. Community leaders should know why it is important that people who are HIV-positive should start taking treatment. […] I think that traditional leaders, pastors, and councillors. And mothers…women who look after virgin girls. Also those who do home based care (referring to CCGs) because they are community members […] People trust community members. They know that if certain people speak, they believe that they are not misleading them. […] I think they can get more people, some will take it seriously. Even the teachers in schools can speak with children, and say that there is something like this (referring to HIV care). If you know someone at home, you have to tell him/her [to access HIV care]. It’s important.” (IDI TasP Nurse 01)

#### Efforts in rethinking transversally the overall health system

HCPs supported the government efforts to integrate HIV services as part of a comprehensive health package (South African model of an “IDEAL clinic” [29]), noting how this would benefit large-scale implementation of universal ART. They were in favour of moving away from a restricted number of nurses specifically caring for people living with HIV, to a nursing workforce who is fully capable of caring for HIV and ART initiation (NIMART-trained) and other diseases. They explained how such health system organization would increase confidentiality for patients accessing the clinics for their HIV infection.

“You will see that this person doesn’t come for regular check-ups for CD4 count. Maybe some reasons are a long waiting time that we have in clinics. The services are divided, like when you came to see me, after we are done I will refer you to ART department, where you will be taking viral load. I think that was the main problem. It’s a long waiting time, joining another queue, and again join another queue to open a clinical chart, and taking of bloods. And another thing is stigma. That I am from this community, and the people from this community will see that I started at the main clinic. Now that I am referred to ART department that means I tested positive. Maybe these were the main things.” (DoH Gov. clinic manager 01)

“The government wants to end this thing of having people who are seeing inclusively. He wants [to implement] a service point for chronic diseases to offer all services to the participant at the same time. So there cannot be a situation where we see someone belongs to ART, separately from other chronic cases. |…] So every nurse should have NIMART. Therefore wherever a participant enters, s/he will find a nurse that will be able to help him/her even if s/he is ART. The nurse will check bloods, and see that it is important to do this, everything you see in a well-trained nurse to work under any conditions.” (FGD TasP Nurse, P2)

According to HCPs, allowing all nurses to provide care for HIV and also for other diseases which are of concern to people living with HIV (e.g. diabetes, cardiovascular diseases) would participate in answering people’s healthcare expectations.

## Discussion

In this high HIV prevalence and rural South African setting, HCPs reported an overall good experience of delivering ART early in the course of HIV infection, both in the government clinics (ART at a CD4 count ≤500 cells/μL) and in the TasP trial (universal ART), with many similar experiences between the two settings. Benefits of enlarging ART use to individuals with high CD4 count for the sake of people’ health were well understood by HCPs, who were glad to report saving people’s life and giving hope to their patients, as also reported by HCPs from high-income countries [12, 13]. HCPs also liked providing counselling to people with high CD4 count who had more ability to concentrate and compared to the sickest ones. According to the HCPs, patients were overall willing to initiate ART before being symptomatic and being seen as sick, which has also been observed in a study conducted in Swaziland [30]. High rates of ART initiation were indeed observed among ART-naïve individuals accessing the TasP clinics [17, 31]. These positive results are encouraging for the scale-up of universal ART in South Africa and elsewhere.

Our results also showed how universal ART changed the perception of HIV treatment among HCPs and their patients. While HCPs used to ask themselves whether a patient was eligible or not to start treatment based on clinical data, they now needed to shift their mindset to better understand their patients’ global characteristics and develop strategies to support them to start treatment. Offering ART early and systematically in the course of HIV infection indeed seemed sometimes challenging for HCPs, with patients who did not feel sick, who did not see HIV care as a priority or needed time to accept and disclose their HIV status and to be ready to engage in HIV care and treatment for life. Reluctance to initiate ART as early as possible was also recently observed in Uganda among high-risk women who were offered universal ART [32]. While HCPs are convinced of the benefits of early ART initiation, they underlined the importance of not forcing people with high CD4 count to initiate ART immediately in order to avoid defaulting and thus offer individualized holistic care [12, 16]. In this context, universal ART thus emphasizes HCPs’ responsibilities in terms of HIV counselling and ART education that should be offered at the first clinic visit but also continuously, and should be adapted according to patients’ needs and behaviors [33]. HCPs especially need to take time to specifically explain the benefits of early ART initiation to their patients who might not be informed of the rapid changes of ART guidelines. HCPs also underlined the need for social support from the family and more generally from the community to increase early access to HIV care and treatment. People with high CD4 count may be particularly motivated to access HIV care seeing other community members being fine while on ART. The involvement of peers as well as community and traditional leaders have also been identified as opportunities to increase HIV awareness within the community and encourage people, especially the men and the youngest individuals, to access clinics and receive HIV care and treatment early in the course of their HIV infection [18].

Furthermore, our results highlighted the need to strengthen existing models of HIV care for the scale-up of universal ART and especially the clinical management of the patients. HCPs interviewed in our study foresaw an increase of people in clinics with the implementation of universal ART, as also suggested by modelling studies [24, 25]. They thus claimed for more human resources, especially NIMART nurses with counselling skills, to be employed to ensure good quality of care [34]. HCPs also underlined the importance of ensuring the availability of drugs in order to avoid ART shortage. HCPs also emphasized that the implementation of universal ART will require substantial efforts to guarantee effective monitoring of people on ART and rapidly detect defaulters, which is key to inform differentiated care and maintain community viral suppression. Yet, while routine viral load testing is essential to detect early signs of ART adherence problems before immunological decline [35], and is now recommended as a standard of care for monitoring [36], such technology is not yet always available. This is not necessarily the case in South Africa but holds in many low and middle income countries [37].

Our results underlined that universal ART is not just giving drugs, but is also an opportunity to rethink the health care system organization. The experiences and perceptions of HCPs highlighted that, without proper support for linkage and for retention, universal ART was unlikely to succeed. While several strategies are known to be effective to increase access to HIV care, they have not routinely been implemented yet in our study setting, one of the most affected by HIV. HCPs thus provided valuable insights as to the state of implementation of these key strategies which could and should be rapidly scaled-up. First, HCPs argued for the delivery of HIV care combining both facility-based and community-based services, aiming at bringing health services closer to where people live or work, and aiming at addressing several barriers to HIV care access, such as distance to clinic. To date, community-based ART programmes have been mainly offered after ART initiation as a key to sustain ART adherence and retention in care [38, 39]. They could also be considered for ART initiation, at home for example [40], although the impact of such an intervention on long-term HIV follow-up is not yet known. The use of SMS reminders and the development of a tracking system including phone calls and home visits by community caregivers, as experienced in the TasP trial, have also been evaluated as effective to link [41] and retain people in care [42]. Secondly, as part of the differentiated ART delivery approach recommended by the WHO [43] aiming at simplifying HIV services and addressing the diversity of needs of people in care and treatment [44, 45], the delivery of 3-month ART supplies for all stable individuals should be facilitated in all clinics. Thirdly, HCPs also reported on the importance of reducing stigma in health care system, through the integration of HIV services within general primary care services, and especially with other long-term conditions, as reported in another study conducted in another rural setting in South Africa [46]. In this context, the South African government recently initiated the “Ideal clinic” programme [29], with the integration of HIV services as part of a comprehensive health package. This programme has not yet been evaluated but certainly provides more and new care opportunities for people living with HIV who do not see their infection as a priority. The wide scale implementation of integrated models of care would nevertheless need investments in training and capacity building support regarding multiple diseases and co-morbidities.

We acknowledge some limitations regarding this study. First, TasP HCPs reported their experiences of delivering universal ART in specific trial clinics, thus not quite in real life conditions. In addition, DoH HCPs were interviewed on their perceptions regarding universal ART, while they had not provided it at the time of the interviews. However, the declarations of both TasP and DoH HCPs regarding the provision of ART early in the course of the infection, and the perceptions of the universal ART scale-up were concordant. Secondly, among participants, only two males (one in the TasP counsellors FGD and one in the TasP nurses FGD) shared their experiences; the sample is however representative of the current gender distribution in the South African health facilities [47]. Third, the FGDs and IDIs were conducted in isiZulu then translated in English, a process that could have led to some loss of information; all the translations were however double-checked by the qualitative interviewer supervisor. Finally, this analysis is specific to a rural South African setting; further research in other settings, elsewhere in Africa or maybe in less HIV prevalent areas, would contribute to fully apprehend the challenges of providing universal ART. The main methodological strength of this study is that there was a strong collaboration for creating the codebook between the qualitative interviewers (who collected, transcribed and translated the data from English to isiZulu) and the two main scientists (who analyzed the data).

In conclusion, while HCPs reported globally good experiences of offering ART at high CD4 counts, this study reflected specific challenges regarding its acceptation by the patients who do not feel sick and not see the value of initiating ART early or might need more time to accept and disclose their HIV status and to be ready to initiate ART for life. In this context, it is essential for HCPs to have appropriate skills and enough time with their patients to counsel them regarding early ART initiation. Insufficient and delayed linkage to care and treatment is problematic in the era of prompt and universal ART; further, if people are not on ART and are not virally suppressed, their risk of HIV transmission is high, thus contributing to sustained HIV incidence and fuelling the epidemic at population level, as demonstrated in the ANRS 12249 TasP trial [31]. Our results confirm that universal ART is more than an increase in ART threshold eligibility and raises challenges for patients, HCPs and decision-makers. It is crucial to continue developing, evaluating, and implementing innovative models of care in order to increase early linkage to and retention in HIV care and treatment and achieve the UNAIDS 90-90-90 target for UTT [48].

## Supporting information

S1 Table Analysis matric on experiences and perceptions of health care professionals regarding universal ART. Hlabisa sub-district, South Africa, 2016.
(DOCX)
