# Supplementary material for: Implementing universal HIV treatment in a high HIV prevalence and rural South African setting – Field experiences and recommendations of health care providers
Source: PLoS One. 2017 Nov 20;12(11):e0186883. doi: 10.1371/journal.pone.0186883 (PMC5695789; doi:10.1371/journal.pone.0186883)
Supplement: S1 Table — Hlabisa sub-district, South Africa, 2016. (DOCX) [file pone.0186883.s001.docx]

**Supplementary file**

*Note that the socio-demographic description of the study population is available in the paper, Table 1.*

**Table S1. Theme 1. HCPs are motivated to provide universal ART, which contributes to better health and better care for people living with HIV *(Hlabisa sub-district, South Africa, 2016)***

|  | **Early/Universal ART revitalized the community** | **Early/Universal ART encourages people to seek care** | **Early/Universal ART allows for improvements of health care delivery** |
| --- | --- | --- | --- |
| **IDI TasP Nurse Manager 01** |  | People who start ART: pioneers  Individual benefits to initiate ART before having symptoms |  |
| **IDI TasP Nurse Manager 02** | Reduction of mortality within the community |  |  |
| **IDI TasP Nurse 01** | Reduction of opportunistic infection | Individual benefits to initiate ART before having symptoms |  |
| **IDI TasP Nurse 02** |  | Individual benefits to initiate ART before having symptoms and being seen sick by the community |  |
| **IDI TasP Nurse 03** | Reduction of opportunistic infection  Reduction of the number of people dying in the same family |  |  |
| **IDI TasP Nurse 04** | Reduction of opportunistic infection | Individual benefits to initiate ART before having symptoms and being seen sick by the community |  |
| **FGD TasP Nurses** |  | Individual benefits to initiate ART before having symptoms (P7, P1) | Easier relation with the patients and planning schedule (P1) |
| **IDI DoH Gov. Clinic Manager 01** |  | People who start ART: pioneers  Individual benefits to initiate ART before having symptoms | Reduction of the burden on health care professionals |
| **IDI DoH Gov. Clinic Manager 02** | Reduction of mortality within the community |  |  |
| **IDI DoH Nurse 01** | Reduction of opportunistic infection |  |  |
| **IDI DoH Nurse 02** | Reduction of opportunistic infection |  | Easier relation with the patients |
| **IDI DoH Nurse 03** | Reduction of the number of very sick people |  |  |
| **IDI TasP Counsellor 01** | Increase of hope in the community | Individual benefits to initiate ART before having symptoms and being seen sick by the community |  |
| **IDI TasP Counsellor 02** | Reduction of the number of very sick people  Increase of hope in the community |  |  |
| **FGD TasP Counsellors** |  | Individual benefits to initiate ART before having symptoms |  |

*TasP: Treatment as Prevention trial; DoH: Department of Health; ART: antiretroviral therapy*

**Table S2. Theme 2. HCPs perceive challenges in providing universal ART to reluctant patients and without sufficient human resources and equipment *(Hlabisa sub-district, South Africa, 2016)***

|  | **Early/Universal ART is offered to people who can be reluctant to initiate ART** | **Early/Universal ART is offered to people who may be more likely to default** | **Early/Universal ART could suffer from human resources and equipment shortage** |
| --- | --- | --- | --- |
| **IDI TasP Nurse Manager 01** | Not feeling sick |  | Fear of not enough human resources  Fear of ART shortage |
| **IDI TasP Nurse Manager 02** | Importance of counselling |  | Fear of not enough human resources |
| **IDI TasP Nurse 01** | Not feeling sick  Fear of engaging in ART for life  Importance of counselling | No disclosure because not feeling sick | Fear of not enough human resources  Fear of ART shortage |
| **IDI TasP Nurse 02** | Not feeling sick, especially the youngest  Fear of engaging in ART for life  Lack of knowledge about early ART | Initiating without having felt sick, especially the youngest  No disclosure because not feeling sick, especially the youngest  Importance of support | Fear of not enough human resources |
| **IDI TasP Nurse 03** | Lack of knowledge about early ART  Importance of counselling | Initiating without having felt sick  No disclosure because not feeling sick | Fear of not enough human resources |
| **IDI TasP Nurse 04** | Not feeling sick  Lack of knowledge about early ART |  |  |
| **FGD TasP Nurses** | Lack of knowledge about early ART (P3) | Initiating without having felt sick: ART not a priority (P2)  No disclosure (P1)  Importance of continuous counselling (P2) | Delays for lab tests results (P1) |
| **IDI DoH Gov. Clinic Manager 01** | Lack of knowledge about early ART  Not feeling sick | No disclosure | Fear of not enough human resources  Fear of ART shortage |
| **IDI DoH Gov. Clinic Manager 02** | Fear of engaging in ART for life  Importance of counselling |  | Fear of not enough human resources |
| **IDI DoH Nurse 01** | Importance of counselling |  |  |
| **IDI DoH Nurse 02** |  | No disclosure | Fear of not enough human resources  Delays for lab tests results |
| **IDI DoH Nurse 03** | Not feeling sick, especially the youngest  Importance of counselling | No disclosure | Fear of not enough human resources  Fear of ART shortage |
| **IDI TasP Counsellor 01** | Fear of engaging in ART for life  Importance of counselling |  | Fear of not enough human resources |
| **IDI TasP Counsellor 02** | Not feeling sick  Fear of engaging in ART for life  Importance of counselling |  | Fear of not enough human resources |
| **FGD TasP Counsellors** | Not feeling sick  Lack of knowledge about early ART |  | Fear of not enough human resources  Fear of ART shortage |

*TasP: Treatment as Prevention trial; DoH: Department of Health; ART: antiretroviral therapy*

**Table S3. Theme 3. HCPs suggest opportunities for successful scale up of universal ART *(Hlabisa sub-district, South Africa, 2016)***

|  | **Efforts in constituting a wide and efficient workforce** | **Efforts in offering a diversity of ART supply strategies** | **Efforts in integrating universal ART in a more comprehensive approach** | **Efforts in rethinking transversally the overall health system** |
| --- | --- | --- | --- | --- |
| **IDI TasP Nurse Manager 01** | Enough human resources |  |  | Integration of HIV services to decrease lack of confidentiality |
| **IDI TasP Nurse Manager 02** | Enough human resources | Allowing ART pick up by community members on behalf of patients  Tracking system with home visits | Community-awareness campaigns  Involvement of community leaders |  |
| **IDI TasP Nurse 01** | Enough human resources | Community-based services | Community-awareness campaigns  Involvement of community leaders | Integration of HIV services to decrease lack of confidentiality |
| **IDI TasP Nurse 02** |  | 3 months ART supply  Community-based services  Tracking system with SMS and phone calls |  |  |
| **IDI TasP Nurse 03** | Enough human resources | Allowing ART pick up by community members on behalf of patients | Community-awareness campaigns | All nurses NIMART trained |
| **IDI TasP Nurse 04** | Adequate training |  |  |  |
| **FGD TasP Nurses** | Adequate training (P4) | 3 months supply (P1, P2)  Community-based services (P2, P6)  Allowing ART pick up by community members on behalf of patients (P5)  Tracking system with phone calls and home visits (P6)  Opening clinics on Saturday (P5) |  | Integration of HIV services to decrease lack of confidentiality (P2, P3)  All nurses NIMART trained (P2, P6) |
| **IDI DoH Gov. Clinic Manager 01** | Enough human resources | Tracking system with SMS |  | Integration of HIV services to decrease lack of confidentiality |
| **IDI DoH Gov. Clinic Manager 02** | Enough human resources | Allowing ART pick up by community members on behalf of patients |  | Integration of HIV services to decrease lack of confidentiality |
| **IDI DoH Nurse 01** | Enough human resources  Adequate training | 3 months supply  Tracking system with SMS  Community-based services | Media mobilization | Integration of HIV services to decrease lack of confidentiality |
| **IDI DoH Nurse 02** | Enough human resources | 3 months ART supply  Allowing ART pick up by community members on behalf of patients |  |  |
| **IDI DoH Nurse 03** | Enough human resources | 3 months ART supply  Community-based services |  | Integration of HIV services to decrease lack of confidentiality |
| **IDI TasP Counsellor 01** | Enough human resources  Adequate training | Community-based services  Tracking system with phone calls |  |  |
| **IDI TasP Counsellor 02** | Enough human resources  Adequate training |  |  |  |
| **FGD TasP Counsellors** | Enough human resources | Community-based services (P5) | Community-awareness campaigns (P2)  First counselling with fieldworkers during home based testing (P1) | Integration of HIV services to decrease lack of confidentiality (P3) |

*TasP: Treatment as Prevention trial; DoH: Department of Health; ART: antiretroviral therapy*
